# Qualitative study of willingness and demand for participation in decision-making regarding anticoagulation therapy in patient undergoing heart valve replacement

**DOI:** 10.1186/s12911-022-01780-2

**Published:** 2022-02-18

**Authors:** YaNing Zang, ShanShan Liu, YuHong Chen

**Affiliations:** 1 The Third School of Clinical Medicine, Nanjing Medical University, Qinhuai District, Nanjing City, Jiangsu Province China; 2 Department of Cardiothoracic Surgery, Nanjing First Hospital, Nanjing Medical University, Qinhuai District, Nanjing City, Jiangsu Province China; 3 Department of Nursing, Nanjing First Hospital, Nanjing Medical University, Qinhuai District, Nanjing City, Jiangsu Province China

**Keywords:** Valve replacement, Decision-making, Anticoagulation, Nursing, Qualitative study

## Abstract

**Background:**

Promoting patient participation in decision-making aims to maintain the partnership between doctors and patients, reflect the patients’ goals, values, and preferences, and achieve patient-centered care. Realizing patient-centered care, shared collaboration between doctors and patients, and the decision-making process that considers the patients’ priorities and goals are the keys to high-quality health care. Therefore, it is indispensable to analyze the patients’ willingness to participate in the decision-making process and related participation needs regarding anticoagulation treatment for patients undergoing valve replacement.

**Purpose:**

To analyze the patients’ willingness to participate in the decision-making process and the participation needs of patients undergoing mechanical cardiac valve replacement in the process of anticoagulation therapy to provide a basis for promoting patients' participation in decision-making.

**Methods:**

Using phenomenological research methods, data were collected through semistructured interviews. Patients were interviewed after mechanical valve replacement from June to August 2021 in a Grade A hospital in Nanjing. Data were analyzed according to the Colaizzi phenomenology method.

**Results:**

Three major themes were identified from the data: strong willingness to participate but low actual participation, supportive needs, and family members’ participation.

**Conclusions:**

This study guided interventions to encourage patients who underwent heart valve replacement to participate in the decision-making process. From the patient's perspective, obtaining support in the decision-making process and caregiver enthusiasm is important. This study prompted thoughts about the use of auxiliary tools and provided a reliable basis for constructing decision-making auxiliary programs to guide clinical practice.

## Background

### Introduction

Heart valve replacement is a routine procedure for the treatment of moderate to severe valvular heart disease and is known to increase the patients’ survival rate [1]. Usually, patients require lifelong anticoagulation therapy after mechanical heart valve replacement to prevent thrombosis and embolism [2]. Currently, new anticoagulation drugs are gradually introduced in the clinic, but the efficacy of the best anticoagulation treatment is uncertain, and the choice of anticoagulation schemes is ambiguous. Choosing the optimal anticoagulation schedule requires comprehensive consideration of the patient's overall cognition, values, disease treatment, and factors such as preference and disease condition [3, 4]. The type of anticoagulation therapy has an important impact on the effects of anticoagulants, and when Posed with the risk of bleeding and thrombosis, patients often experience conflicts in decision-making [5, 6]. Participating in the decision-making process means that patients partake in their disease awareness, provide disease information, understand their views on treatment, and actively participate in the treatment process [7]. The risk of complications after mechanical valve replacement, complications from medications, dietary requirements, and continuous follow-up all impose a major burden on patients. This also highlights the response to the patient's care needs for participation in decision-making and improves the degree of patient participation in decision-making to achieve the best patient outcomes. Therefore, it is necessary to encourage patients with mechanical valve replacement to participate in decision-making, and an in-depth understanding of patients’ willingness and need to participate in the decision-making process is an important prerequisite. Qualitative research is increasingly applied to independent or mixed-method research. It is a research method that emphasizes patient-centered research. It facilitates obtaining an understanding of patients’ experiences, values, and priorities, reveals their value preferences, and supports clinical practice. The key to high-quality health care is information sharing and collaboration between doctors and patients, combined with a decision-making process that completely considers the patients’ priorities and goals [8, 9]. The purpose of this study was to elicit the opinions, value preferences, and priorities of patients with valve replacement, to obtain their needs for participating in treatment decision-making, to enable better joint decisions for their care, and to provide a basis for clinical nursing practice.

### Literature review

As the medical model changes and the concept of “patient-centered” care increasingly become acceptable, the role of patients in the entire disease management process is receiving increasing attention, especially the power of patient participation and informed consent. Laws, regulations, and professional norms are becoming mandated [10]. In 2014, the WHO called on patients to actively participate in clinical decision-making, to ensure the rights of patients to participate in their own treatment plans, to promote humanistic care, and to maximize the benefits of treatment [11]. Decision-making participation means that patients are actively engaged in their own treatment decisions. It is a process through which clinicians and patients discuss various options, benefits, and adverse effects while considering the patient's value, goals, preferences, etc. to make joint health decisions [12]. There is increasing evidence that patient participation in treatment decision-making has a positive effect on their outcomes. Studies have found that the participation of patients with atrial fibrillation in decision-making on anticoagulation therapy can help them to understand relevant information, form rational preferences, relieve their anxiety, and improve clinical outcomes [13]. Studies have shown that there is a significant increase in the understanding of the benefits and harms of anticoagulant therapy in elderly patients who engage in the decision-making process, which can completely improve patients' compliance with treatment [14]. Although patient participation in decision-making has been proven to have positive significance in improving prognosis, it is challenging. According to a survey of the decision-making experience in the prevention of cardiovascular diseases in the elderly [15], most patients have only a few people who support and completely understand the purpose, potential benefits, and risks of patient participation in decision-making. Participants have major differences in cardiovascular disease-related goals and preferences, which influences patient participation in decision-making. The Ottawa decision support framework (ODSF) is a practical and neutral theoretical framework based on multidisciplinary theory to promote patient participation in decision-making [16]. The primary premise of ODSF is to assess the patient's decision-making needs, that is, whether there are decision-making conflicts, insufficient decision-making knowledge, unclear values, social support, etc., to implement interventions based on needs to achieve a positive outcome. This theory is mainly applied in cancer care, cardiovascular disease, mental illness, and other fields [17]. At present, there is limited research on the participation of patients with mechanical valve replacement in decision-making, and the supportive needs of this patient group during the entire treatment with anticoagulants are poorly understood. Therefore, this qualitative study, based on ODSF theory, assessed the relevant information requirements and support situation of patients’ participation in decision-making through interviews. Moreover, it also comprehensively considered patients’ values and preferences in guiding and improving the patients’ ability to rationally participate in decision-making.

## Methods

### Qualitative approach and research paradigm

This article was written as follows to the SRQR guidelines [18], and this research is based on the descriptive phenomenological method. The term "phenomenology" was introduced by the German philosopher Husserl. Descriptive phenomenology, also known as transcendental phenomenology, is a qualitative research method developed by Husserl. There are three research processes in this method: intuition, phenomenon analysis, and description [19]. It refers to the analysis and study of the internal and external components of the individuals’ situation, the refinement of the elements, and the exploration of each. It explores the relationship between the elements and the environment to obtain an in-depth understanding of life experiences [20]. This method can enable researchers to deeply understand the relevant participation needs of the research subjects.

### Informants and setting

This research used a purposeful sampling method because it was anticipated that additional relevant information could be obtained. Patients with mechanical valve replacement in a third-class hospital in Nanjing from June to August 2021 were selected as the research subjects. Inclusion criteria were patients with mechanical valve replacement who needed anticoagulation therapy after surgery, aged 18 years or older, had a certain understanding and expression ability, provided informed consent, and could cooperate with the study. The exclusion criteria were severe heart, liver, or kidney disease; a history of mental illness; communication difficulties and inability to cooperate to complete the study; and persons with contraindications to anticoagulants complicated by a peptic ulcer, bleeding tendency, anemia, or a history of previous bleeding. In this qualitative study, there was no fixed sample size or criteria. The main basis was data saturation, implying collecting data until no new information was deemed available.

### Data collection

Data were collected through semistructured interviews, and the interview outline was prepared before the interview as follows:

1. What is your understanding of the anticoagulant treatment plan after valve replacement?
2. What do you understand about decision-making participation? Have you participated in your treatment decisions before?
3. What do you think are the benefits of patient participation in decision-making? Are you willing to participate in treatment decisions?
4. In the process of participating in decision-making for anticoagulant therapy, do you have any decision-making difficulties or contradictions?
5. In your opinion, what type of support do you need from medical staff to support the decision-making process for anticoagulant treatment, whether it is intellectual, psychological, or other aspects?

In addition, the researchers also used guiding methods, such as "Can you tell me more about this? Do you have anything else to add?" To obtain additional valuable information, an interview outline was used during the interview. The interviews were based mainly on but were not completely limited to the structure of the outline.

Before the interview, the purpose, content, methods, and presentation of the results were explained to the patient. All participant interviews were audio-recorded. After obtaining the patient’s consent, the recording was made, and the patient was reassured that the data would be used only for this study, their privacy would be protected, and their names would be replaced with codes. The interview was conducted in a quiet and independent ward, and data collection and analysis were conducted simultaneously until the data were saturated and no new themes appeared. The duration of the interview was 20 to 45 min. The interviewer encouraged the interviewees to completely express their thoughts and needs by listening carefully and using interview techniques such as questioning, listening, and responding promptly. Body language can convey several types of messages simultaneously; messages may be conveyed through facial expressions, gaze, gesture, body posture, foot and leg movements, etc. During the interview, the patient’s facial expressions and body language were simultaneously recorded. The recordings were converted into textual materials within 24 h after the end of the interview. If there were any doubts or unclear details, the research subjects were contacted for reconfirmation.

### Data analysis

The interview results of this study were analyzed by Microsoft Word and Excel. Data analysis was based on Colaizzi’s 7-step analysis method of phenomenological data [21]. First, within 48 h after the interview, two researchers sorted the interview materials. The researcher listened to the recordings repeatedly and transcribed them into text version, sentence by sentence. The ambiguous parts in the recordings were highlighted and verified by the interviewees within 24 h. At the same time, the researcher identified facial expressions, body movements, and postures as supplementary material. Second, two researchers extracted statements that were meaningful and consistent with the research phenomena and recorded them in a word document. Then, the researchers summarized and extracted the meanings from the meaningful statements. These meaningful statements were classified and sublimated to develop thematic concepts such as participation, supportive needs, and family participation. Third, the researchers found common features or concepts of meaning, forming themes, clusters of themes and categories, and linked themes to research phenomena and provided a complete narrative. Finally, one of the researchers integrated the results and described the research phenomena in detail to state the essential structure of the phenomenon. The results of the data analysis were integrated into the detailed content of the patients’ participation in the decision-making process. In the translation phase, we translated the results into English after the analysis phase, and all the results were reviewed by native English speakers. We acknowledged our preconceived ideas on the study topic and adopted an open attitude of learning from the informants’ personal experiences.

### Ethical considerations

Ethics approval was obtained through the Ethics Committee of Nanjing First Hospital. The hospital affiliated with Nanjing Medical University undertakes its ethical review. Participants provided written informed consent before the interview. During the interview, the respondent’s condition could change, and the survey did not pose a threat to their safety and health. The subjects did not bear any financial burden during the research process. Participants were assured that the research results would be published in different ways, but no relevant information would be displayed, and the privacy of the participants would be respected and protected.

## Results

The study was conducted from June to August 2021 and recruited 12 patients and their primary caregivers. They were between 20 and 66 years of age and included 8 males and 4 females. All patients underwent mechanical heart valve replacement surgery, and there was no significant difference in the general information of the patients. Three themes were identified from the data: participation in decision-making, supportive needs for participation in treatment, and participation of family members.

### Participation in decision-making

#### Strong willingness to participate in decision-making

Most patients expressed willingness to actively participate in their own anticoagulant treatment decisions when asked. Interviewee 2: "It is beneficial to participate, and we are willing. I hope to have a clear understanding of the pros and cons of the treatment, the precautions after the operation, and how it is good or bad for my health. You know.” The study also found that patients tended to participate in a collaborative relationship with doctors. Interviewee 1: "Of course, I am willing to participate. I am an old patient and have stayed in several hospitals before. I truly want to communicate my thoughts with the doctor. More communication with the doctor will be better to "Know your condition", but some interviewees expressed that they were unwilling to participate. This may be related to a lack of knowledge to completely adhere to the doctor's advice. For example, interviewee 3: “We don’t understand anything. I’ll do whatever the doctor asks me to do. It’s up to your arrangements.”

#### Actual participation was low

Although most patients expressed their desire to actively participate in treatment decision-making after their valve replacement, they and their families did not know how to participate and mainly followed the doctor's arrangements. Interviewee 2 said: "In this regard, you doctors and nurses must be professionals. You have seen so many patients and have taken care of them for so long. You have your procedures, and we will just follow your arrangements." The influence of attitude. Interviewee 4: "How do I participate? Doctors and nurses are usually very busy, time is limited, and no one comes to teach me how to do it." In addition, the study also found that most patients had misunderstandings about decision-making participation. Interviewee 6: "We don’t know what participation means. The doctor talked about the pros and cons of the treatment and precautions before and after the operation. This must have been told to me, and I have been involved. Got it."

### Participation in the supportive needs of treatment decision-making

#### Information needs

Treatment information is the basis of treatment decision-making. Patients have a wide demand and excess literature for information on anticoagulant therapy. During the interview, patients focused on the side effects of drugs, the precautions, related matters for review, and the pros and cons of anticoagulant treatment and were eager to receive support and help from medical staff. Interviewee 8: "I need to know what is best for me and what is bad for me during such a major operation. I need to concentrate on is how I should focus on diet and exercise after the operation. What side effects do anticoagulants have on me, and which type of medicine is best for my condition, I am not sure which to choose.” Patients had limited access to information, mainly through online searches or advice from friends. Interviewee 3: "We have a relative who just had an operation here and was discharged from the hospital. I asked him about how to review after discharge, how to draw blood, and how to look at this test. It is more convenient to search on the internet, and I know too little. I still hope that your medical staff can tell us more.” Different patients focus on information due to their disease conditions. For example, respondent 7: "I am a patient with hypertension. Does taking anticoagulants have any effect on blood pressure? Do I need to take antihypertensives, and should I be separated from my caregiver?" Respondent 6: "I am afraid of being discharged from the hospital. Is there any effect on my menstruation? I had irregular menstruation before. Will taking this medicine worsen menstrual bleeding and what effect will it have on me?" Most patients have a strong willingness to acquire knowledge and information, while a few patients expressed the need for long-term continuous information support. For example, interviewee 4: "We need a doctor to provide a detailed explanation of anticoagulants, contraindications, complications, and precautions. It is best to have a brochure or something online that can remind me; otherwise, I’m afraid I’ll forget it for a long time.” Interviewee 10: “I had hypothyroidism (hyperthyroidism) before. Do potassium-containing foods need to be contraindicated, and if I catch a cold, will taking cold medicine affect warfarin? Can you give me a paper list of contraindications in diet and medication? I cannot remember much of the doctor’s advice."

#### Psychological support needs

Patients with heart disease are usually overweight or obese and prone to anxiety and fear after undergoing the traumatic stress of major surgery. During the interview, most patients expressed doubts and concerns about their normal life and quality of life after being discharged from the hospital. Interviewee 3: "I also want to be clear about the overall long-term development of the disease, so that I have a general understanding of my disease. Can I live a normal life? I knew that the use of mechanical valves must be treated with lifelong anticoagulants, but I do not know if it will affect my life. I am still quite worried." Interviewee 5: "I am still very young. Will it affect my work and life after being discharged from the hospital? Can I live like a normal young man?" Some of the patients also felt guilt and burden towards their families. Interviewee 9: "I have spent large amounts of money in the hospital for so long, and I have to take medicine all the time after I leave the hospital, which places such a heavy burden on my family. Sometimes I hate myself for being unwilling to live with such a disease." Interviewee 11: "After I was discharged from the hospital, I still need to take blood tests to see what indicators are truly worrisome. I must go to the hospital every two days. I don’t want to spend money, and my family is also affected. I still have a burden in my heart. Yes." In addition to the patient's psychological pressure, the patient's family members are mentally stressed and depressed. For example, interviewee 12 stated, "The warfarin that I have taken for so long after being discharged from the hospital has serious side effects. Heart surgery is a major operation. My family is very nervous.” He was afraid of poor drug control and frequent hospitalization. “I'm going to be sad again."

#### Patients' value preferences

Patients will participate in irrational ways based on their empirical judgments and intuitions, which may cause conflicts in decision-making. For example, respondent 7 said, "Because I am not a local, it is quite troublesome to come here. I want to know if I can come here for the discharge review and check-out at our local hospital. The operation has been completed. It should be easy to determine." The patient's social support also affected their attitude towards treatment and preferences. For example, interviewee 10 said, “This hospitalization has already cost a large amount of money, and long-term anticoagulant treatment is required after discharge. What will be the consequences if it is interrupted? There is also a need to take medicine and review it all the time. I do not know if I can talk to the doctor. Can I discuss with the doctor to change to an inexpensive medication?” Some of the interviewees were influenced by cultural or media propaganda and asked to use foreign drugs.

Interviewee 6: “Is there any difference between imported and domestic medicine? Does the imported medicine have fewer side effects? Then, I have to tell the doctor that I want to use imported medicine.”

#### Trust in medical staff

There exists a knowledge asymmetry between doctors and patients, with doctors considered the source of medical expertise concerning patients’ health. Furthermore, a busy clinical environment will affect a patient’s access to health information and affect decision-making participation. In the interview, the interviewees expressed that they had a high degree of trust in medical staff and were eager to have more opportunities for direct communication with them. Interviewee 5: "We can understand that doctors and nurses are very busy, but we also want you to stand on the perspective that patients need consideration. Our people believe in you, and we will not believe in what others say because you are all professionals. I hope you can talk to us more and communicate more.” The interview also found that patients believed that nurses could help them understand treatment-related information and prepare for decision-making and participation. For example, interviewee 6: "We have more opportunities to see nurses. Sometimes we ask nurses questions such as the precautions for taking medicine, how to review them, and nurses know a great deal."

### The participation of family members is essential

Family members are an important part of the patient's social support system and play a vital role in the treatment decision-making process. Family members are the main referees and negotiators in the decision-making process for patients. For example, interviewee 2 stated, "The psychological support of my wife and children during the entire hospitalization is one of my pillars. I have lived to make many decisions, except for listening to the doctor and discussing with my wife and children in the family." It was also found that the family members were the "spokespersons" of the patients. For example, respondent 8: "I am getting older and have a bad memory. Since I was hospitalized, my son has cared for me. The doctors spoke with him, and if I have any problems or discomfort, it is my son who will go to the doctor and come back and tell me." When the patient is too young or too old, family members sometimes become the main decision-makers. For example, respondent 3: “I am still young, only twenty years old. I was very panicked after such a major operation. During hospitalization, my parents were by my side to care for me. The operation and the specific treatment were decided by my parents. After discharge from the hospital, I mainly listened to my parents for specific long-term anticoagulant treatment, and my parents will decide for me how to check and take medicine." Interviewee 12: "I am old; how can I remember so many things? It’s my son who remembers for me, how to take medicine, what needs my attention to, how to take blood for examination, and if there is any problem, he will decide."

## Discussion

To analyze the patients’ willingness to participate in decision-making and related participation needs in the process of anticoagulant treatment for patients with valve replacement. Using phenomenological research methods, data were collected through semistructured interviews. Three major themes were identified from the data: strong willingness to participate but low actual participation, supportive needs, and family members’ participation. The main results are discussed as follows:

1. Medical staff should use decision-making tools to meet the patients’ needs for information.
  Information is the basis of decision-making, and patients can only make rational decisions if they have sufficient and effective treatment information. The survey of the patients’ needs for participation in this study found that patients with mechanical valve replacement lack knowledge, especially the precautions for anticoagulants and related complications. Qualitative research found that patients’ information needs were diverse and that there is an abundant amount of available content, especially as regards disease-related information, anticoagulant medication risks, medication precautions, and review matters. In response to various information needs, medical staff should meet patients' information needs at an early stage and provide a corresponding basis for patients to analyze treatment information to facilitate practical choices. Studies have shown that during the decision-making process and patients’ participation, willingness to acquire knowledge is significant, but the level of acquisition behavior is low [22]. This study found that most patients obtain relevant treatment information through various sources, including internet searches and friendly advice. The challenge for patients with limited access to information is the ability to distinguish between true and false information, and uneven quality of information becomes prominent. A decision-making tool promotes patient participation in decision-making. Its role is to clarify the decisions, provide information about treatment results, and explain how personal values are combined with choices to help patients participate in their health care decisions [23]. Presently, foreign decision-making aids for the cardiovascular specialty are gradually introduced into the clinic. A systematic review of the cardiovascular specialty showed that decision-making aids mainly included providing disease treatment information, analysis of the pros and cons of treatment options, and clarification of patient values [24], aiming to help patients understand the pros and cons of treatment options and their impact on quality of life, as well as to guide patients to think, clarify value orientation, and make satisfactory decisions [25]. Holbrook demonstrated that decision-making aids such as decision boards, decision manuals with audiotapes, or computer programs have significantly improved the patients’ level of knowledge. Studies have shown that the higher the patient's knowledge level, the better the compliance with treatment [14]. Satisfying the needs of patients with valve replacement for anticoagulant knowledge and information is the primary prerequisite for promoting patient participation in decision-making. This suggests that medical staff should adopt high-quality decision-making aids, such as providing patient treatment information with the help of videos, manuals, decision-making guidelines, and networked channels, and provide targeted information support according to the patient's decision-making information needs and concerns. Additionally, the intent of the patient group to participate in decision-making was influenced by the medical staff. We all need to change the previous concept of "everything is according to the doctor" create a healthy atmosphere for communication, and embrace the "patient-centered" medical model.
2. Nurses assist in information support and promote high-quality decision-making participation.
  The busy environment of clinical practice makes it impossible for doctors to provide adequate information. In a study of decision-making assistance, Stacey proposed that nurses act as the personnel who provide positive and neutral guidance to patients during the decision-making process [26]. In this interview study, some patients said that clinical nurses had sufficient medical knowledge and were also a source of reliable treatment information. The study also found that some patients said they could adjust medication upon review. In the final analysis, they had insufficient information about anticoagulation, and they could neither weigh the pros and cons nor make the right choice. Each patient has the right to know and the right to choose. Medical staff should respect the patient's values and preferences, guide patients to participate in the decision-making process, and meet their participation needs. Légaré [27] applied the Ottawa decision support framework as a decision support theory, which mainly included three aspects: assessing the demand for decision support, providing decision support, and evaluating the outcome of decision-making, plus providing patient decision-making guidance and support through clinical consultation and decision-making aids. While meeting the patients’ information needs in clinical practice, nurses should also improve patient education. When educating patients about anticoagulants, attention should be given to the precautions of drug use, such as the dietary precautions of warfarin and the drug-drug interactions. Nursing staff must use different communication skills to enhance the delivery of information to ensure that patients correctly understand relevant information and to eradicate patients' misconceptions. Nurses should also strengthen the interaction with patients and their families based on the Ottawa decision support framework theory, assess patient needs, provide patient participatory care, and assist patients in correctly understanding treatment plans [28]. Additionally, they should provide comprehensive consideration of patient preferences, requests, and needs, use auxiliary tools to provide targeted decision support, guide and assist patients in participating and choosing the best treatment and nursing measures, promote patients’ participation in making high-quality, and achieve high-quality medical care.
3. Focus on the emotional changes in the patient's decision-making process and provide psychological care.
  Heart valve replacement surgery and life-long anticoagulant therapy are major causes of stress, which can cause patients to produce an anxiety-based physiological stress response [29]. The interview determined that some patients were nervous and anxious when they were first admitted to the hospital, especially for heart diseases, which are usually serious and require more attention. Studies have shown that patients often experience decision-making conflicts while participating in treatment decision-making; they feel nervous and anxious, and sometimes there are delays in decision-making [30]. Patients with mechanical valve replacement must undergo lifelong anticoagulant therapy. When faced with many treatment options and weighing the pros and cons of the plan, most patients who do not have common medical knowledge will be at a loss, not knowing how to choose and worrying about the prognosis, resulting in negative emotions. Studies have shown that a poor emotional experience also severely affects patient compliance with treatment and physical symptoms and affects the treatment effect of the disease [31]. There are many emotions in the treatment decision-making process for patients with valve replacement, and these emotions may have ramifications such as in causing patients to avoid choices and delay decision-making. In this interview study, it was found that most patients had certain negative emotions. Most of them had hospitalization anxiety, were worried about the prognosis, and had heavy financial burdens that led to high mental pressure. Patients also often experience insomnia and decreased appetite. Clinical nurses inherently have the closest daily contact with patients and can promptly detect abnormalities. Therefore, while performing nursing operations, nurses should learn to identify the patient’s bad mood, communicate with the patient promptly, and formulate appropriate intervention measures, including music therapy, diet intervention, and respiratory therapy, according to the patient’s symptoms to alleviate the patient’s tension and discomfort. Nurses should also have empathy, learn to listen to and understand patients, provide psychological encouragement and comfort, and improve their psychological state to reduce or eliminate patients' negative emotions.
4. Promoting the participation of family members and easing the pressure on patients during the decision-making process.

The patient's family is an intermediary between the patient and the doctor and always plays an important role in the entire process of the patient's treatment. Family members are important assistants in treatment decision-making. On the one hand, they can provide emotional, information, and respectful support [32], which can reduce the patients’ burden and their uncertainty in treatment [33]. On the other hand, as the patients’ spokesperson, family members can uphold the patients’ interests, provide valuable information and help the patient consult and treat related issues [34]. In qualitative research, it is found that family members often analyze decisions together with patients and sometimes even become the main decision-makers, making decisions on behalf of patients. In the interview, it was also found that the family members of the patients wanted to participate in the consultation on treatment because the treatment and rehabilitation of the patients also affected them, especially for patients who needed long-term anticoagulant therapy after mechanical valve replacement, long-term medication supervision, and INR. The participation of family members was essential for such patients. In the decision-making process, medical staff should first evaluate the patient’s social support. A psychological survey of family members related to valve replacement surgery showed that family members have certain psychological disorders during the accompanying period, which can cause fatigue, poor sleep, fear, and other problems [35]. As an important social support system, the participation of family members is a positive effect that cannot be ignored by doctors and patients. The families express the need for emotional support. Chinese cultural beliefs could have influenced families’ emotions and concerns. In clinical nursing practice, families with special cultural beliefs have a great impact on their emotional responses and practical needs. This requires us to focus on the diversified needs of patients, and family members should be given sufficient attention, especially to their psychological problems, with timely guidance and intervention when necessary.

## Conclusion

With the advancement of "patient-centered" health care, the promotion of patient participation in decision-making plays an increasingly important role in clinical practice, and "how to promote patient participation" has become an urgent problem to be solved. The study found that patients with valve replacement need information and content during anticoagulant therapy, and patients need more social support and psychological nursing to improve their prognosis. Influenced by traditional culture, it is still a long way to promote patients’ decision-making participation in the future. This paper offers a direction for patients’ decision-making participation based on the study results.

## Data Availability

The data that support the findings of this study are available from third party name but restrictions apply to the availability of these data, which were used under license for the current study, and so are not publicly available. Data are however available from the authors upon reasonable request and with permission of third party name.

## References

[1] Christensen TD, Skjøth F, Nielsen PB, Maegaard M, Grove EL, Larsen TB (2016). Self-management of anticoagulant therapy in mechanical heart valve patients: a matched cohort study. Ann Thorac Surg.

[2] Li BX, Liu SD, Qi L (2020). Comparison of different bridging anticoagulation therapies used after mechanical heart valve replacement in Chinese patients—a prospective cohort study. J Cardiothorac Surg.

[3] MacIsaac S, Jaffer IH, Belley-Côté EP, McClure GR, Eikelboom JW, Whitlock RP (2019). How did we get here?: a historical review and critical analysis of anticoagulation therapy following mechanical valve replacement. Circulation.

[4] Vaanholt MCW, Weernink MGM, von Birgelen C, Groothuis-Oudshoorn CGM, IJzerman MJ, van Til JA (2018). Perceived advantages and disadvantages of oral anticoagulants, and the trade-offs patients make in choosing anticoagulant therapy and adhering to their drug regimen. Patient Educ Couns.

[5] Huang R, Yang X, Song X, Victor MM, Henry HT (2017). The current status and expectation of shared-decision making in Chinese patients with cardiovascular diseases. Med Philos.

[6] Coffey M, Hannigan B, Meudell A, Jones M, Fitzsimmons D (2018). Quality of life, recovery, and decision-making: a mixed-methods study of mental health recovery in social care. Soc Psychiatry Psychiatr Epidemiol.

[7] Thompson AG (2007). The meaning of patient involvement and participation in health care consultations: a taxonomy. Soc Sci Med.

[8] Morton RL, Sellars M (2019). From patient-centered to person-centered care for kidney diseases. Clin J Am Soc Nephrol.

[9] Guha C, Viecelli AK, Wong G, Manera K, Tong A (2021). Qualitative research methods and its application in nephrology. Nephrology.

[10] Rath D, Nawaz M (2018). Antiplatelet treatment for catheter-based interventions in high-risk patients: current guidelines and expert opinion. Hamostaseologie.

[11] Stacey D, Légaré F, Lewis K (2017). Decision aids for people facing health treatment or screening decisions. Cochrane Database Syst Rev.

[12] Boos CJ, Brown L (2016). Anticoagulation in atrial fibrillation and chronic heart failure: the risk and drug of choice. Curr Opin Cardiol.

[13] Wu Q. Study on process, influencing factors and information processing feature of atrial fibrillation patient engagement in the treatment decision making. Doctoral Dissertation. Naval Medical University of the Chinese People's Liberation Army; 2019 **(in Chinese)**.

[14] Holbrook A, Labiris R, Goldsmith CH, Ota K, Harb S, Sebald RJ (2007). Influence of decision aids on patient preferences for anticoagulant therapy: a randomized trial. CMA.

[15] Jansen J, McKinn S, Bonner C, Muscat DM, Doust J, McCaffery K (2019). Shared decision-making about cardiovascular disease medication in older people: a qualitative study of patient experiences in general practice. BMJ Open.

[16] O'Connor AM, Tugwell P, Wells GA (1998). A decision aid for women considering hormone therapy after menopause: decision support framework and evaluation. Patient Educ Couns.

[17] Yu S, Mou W, Jin Y, Gong J, Liu B, Tan L (2021). Doctor–patient joint decision-making series 2: a model for joint doctor–patient decision-making-Ottawa patient decision aid tool research group. New Med.

[18] O'Brien BC, Harris IB, Beckman TJ (2014). Standards for reporting qualitative research: a synthesis of recommendations. Acad Med.

[19] Naylor MD, Lustig A, Kelley HJ, Volpe EM, Melichar L, Pauly MV (2013). Introduction: the interdisciplinary nursing quality research initiative. Med Care.

[20] Lee TY, Landy CK, Wahoush O, Khanlou N, Liu YC, Li CC (2014). A descriptive phenomenology study of newcomers' experience of maternity care services: Chinese women's perspectives. BMC Health Serv Res.

[21] Colaizzi P, Valle RS, King M (1978). Psychological research as a phenomenologist views it. Existential phenomenological alternatives for psychology.

[22] Cheng ZS, Bai XL, Xie GH, Zhang Z, Lou T (2020). Determinants of participation in treatment decision-making by patients undergoing cardiac valve replacement. J Nurs Sci.

[23] LeRouge C, Nguyen AM, Bowen DJ (2021). Patient decision aid selection for shared decision making: a multicase qualitative study. Med Care Res Rev.

[24] Shi RZ, Gong C, Kang XF (2019). Decision aids clinical application among cardiovascular disease patients: a systematic review. Chin J Mod Nurs.

[25] Mitka M (2013). New guidance for ICD implantation offers decision aids for physicians and patients. JAMA.

[26] Coylewright M, Dick S, Zmolek B (2016). PCI choice decision aid for stable coronary artery disease: a randomized trial. Circ Cardiovasc Qual Outcomes.

[27] Légaré F, O'Connor AC, Graham I (2006). Supporting patients facing difficult health care decisions: use of the Ottawa decision support framework. Can Fam Phys.

[28] Stacey D, O'Connor AM, DeGrasse C, Verma S (2003). Development and evaluation of a breast cancer prevention decision aid for higher-risk women. Health Expect.

[29] Zhan YX, Yu JH, Li SY (2021). Nurses’ cognition on the practice of decision-making by nurses and patients: a qualitative study. J Nurs Sci.

[30] Yang H, Shao Y (2021). Application value of whole process management mode of doctor-nurse integration based on SBAR communication mode in perioperative nursing of heart valve replacement. Value Appl Clin Med Res Pract.

[31] Dharmarajan TS, Varma S, Akkaladevi S, Lebelt AS, Norkus EP (2006). To anticoagulant or not to anticoagulant? A common dilemma for the provider: physicians' opinion poll based on a case study of an older long-term care facility resident with dementia and atrial fibrillation. J Am Med Dir Assoc.

[32] Zhang ZC, Xie FL, Chen AP, Bai LH, Guo YB (2020). A qualitative study of the psychological experience and support needs of patients with advanced lung cancer participating in and implementing treatment decisions. Mod Clin Nurs.

[33] Lamore K, Montalescot L, Unitas A (2017). Treatment decision-making in chronic diseases: what are the family members' roles, needs, and attitudes? A systematic review. Patient Educ Couns.

[34] Hirpara DH, Cleghorn MC, Sockalingam S, Quereshy FA (2016). Understanding the complexities of shared decision-making in cancer: a qualitative study of the perspectives of patients undergoing colorectal surgery. Can J Surg J Can Chir.

[35] Laidsaar-Powell R, Butow P, Bu S (2016). Family involvement in cancer treatment decision-making: a qualitative study of patient, family, and clinician attitudes and experiences. Patient Educ Couns.

